# *Ananas comosus* Peels Extract as a New Natural Cosmetic Ingredient: Oil-in-Water (O/W) Topical Nano Cream Stability and Safety Evaluation

**DOI:** 10.1155/2022/2915644

**Published:** 2022-05-12

**Authors:** Nur Azzanizawaty Yahya, Roswanira Abdul Wahab, Nursyafreena Attan, Mariani Abdul Hamid, Norhayati Mohamed Noor, Rovina Kobun

**Affiliations:** ^1^Department of Chemistry, Faculty of Science, Universiti Teknologi Malaysia, 81310 UTM Johor Bahru, Malaysia; ^2^Enzyme Technology and Green Synthesis Group, Faculty of Science, Universiti Teknologi Malaysia, 81310 UTM Johor Bahru, Malaysia; ^3^School of Chemical and Energy Engineering, Faculty of Engineering, Universiti Teknologi Malaysia, 81310 UTM Johor Bahru, Malaysia; ^4^Cosmeceutical & Fragrance Unit, Institute of Bioproduct Development (IBD), Universiti Teknologi Malaysia, 81310 UTM Johor Bahru, Malaysia; ^5^Faculty of Food Science and Nutrition, Universiti Malaysia Sabah, Kota Kinabalu 88400, Sabah, Malaysia

## Abstract

*Ananas comosus* peels (*Ac*P) are among the agro-industrial biomasses contributing to a significant volume of waste in Malaysia. Thus, the *Ac*P extract (*Ac*PE) may prove useful for other applications, such as an ingredient in a nanocream for controlled delivery for dermal application. Therefore, this study aimed to develop an oil-in-water (O/W) nanocream using ingredients derived from the *Ac*PE and test its stability alongside safety evaluation. The extract is a rich source of polyphenolic compounds *viz.,* catechin, quercetin, and gallic acid. The study discovered that the optimized *Ac*PE nano cream was stable against coalescence during the accelerated test but was influenced by Ostwald ripening over 6 weeks of storage at 4°C. Safety assessments affirmed the *Ac*PE nano cream to be free of microbial contamination and heavy metals. The findings conveyed that the *A. comosus* nano cream is a good cosmetic ingredient and may contribute to the cosmeceutical industry's new and safe topical products.

## 1. Introduction

*Ananas comosus* (L.) Merr is a species in the family of Bromeliaceae which grows well in tropical and subtropical regions, such as Thailand and Malaysia [1]. Due to its pleasant aroma and flavor, the *A. comosus* is usually eaten fresh, and some are processed into *viz.* jams, jelly, juice, and dried products [2]. As the 18th world's largest *A. comosus* producer, Malaysia contributes to the rising amount of discarded biomass which reaches almost ∼1.2 million tonnes/year, consistent with brisk developments in the agricultural sector [3, 4]. This meant that refuse or biomass from this commodity might be put to a good use for other applications. For instance, rather than scouring the depths of forests for exotic plants and exacerbating the overexploitation of our already fragile ecosystems, the polyphenolic-rich *A. comosus* peels extract (*Ac*PE) containing catechin, quercetin, gallic acid, and their derivatives could be a promising and sustainable source of bioactive compounds for rejuvenating the human skin [5, 6].

As a matter of fact, the term “cosmeceutical” was coined in 1961 by the founding member of the US Society of Cosmetics Chemist, Raymond Reed. In the year 1984, Dr. Albert Kligman further used the term by referring to it as a material having both cosmetic and therapeutic advantages [7]. Generally, cosmetic refers to any item that enhances the skin's appearance, boosts the cleansing, and stimulates the skin allure [8]. Noteworthily, the cosmetic industry is among the first to capitalize on nanotechnology-based materials by introducing liposome moisturizing cream in the early 1960s. Since then, nanotechnology in the cosmeceutical industry has grown remarkably and can be considered one of the most competitive technologies of the 21st century [8]. Among the diverse nanoemulsion systems in nanotechnology, such as oil-in-water (O/W) or water-in-oil (W/O) dispersions, both are extensively used in the cosmeceutical industry as carriers for controlled delivery [9]. Also, the physical appearance of nanoemulsions could exist as (1) transparent or translucent (50−200 nm) and (2) creamy (up to 500 nm), both of which are dictated by particle size [10, 11]. Literature has shown that plant-based ingredients in nano cream-emulsified forms are more effective for topical transmission across the skin barrier [12]. Aside from improving the active ingredients' stability, the minute size of the bioactive particulates promotes better penetration of the active ingredients through the skin [13, 14].

In our previous work, the catechin, quercetin, and gallic acid-rich crude *A. comosus* phytoextract formulation *Ac*PE oil-in-water nano cream was optimized, and the physicochemical properties of the produced cream were characterized. Transmission electron microscopy analysis showed that the size of the droplets was in the range of 28.86 to 100.19 nm [15]. This study chose the O/W type nanoemulsion because of its less greasy texture and was likely to incur a lower cost due to the high amount of water in the formulation. Because of this, the nanoemulsion was expected to impart a pleasant after-feel following its topical application on the skin. The formulation was anticipated to spread better over the skin without producing a cream-like coating. That said, this formulation type could aid in hydrating the skin's stratum corneum [16, 17]. To the best of our knowledge, the work proposed in this study is the first-ever attempt to utilize the optimized *Ac*PE nano cream as the bioactive ingredient for topical cosmetic application. Also, results on the accelerated stability, alongside the safety evaluations of the optimized AcPE nano cream, are reported and discussed.

## 2. Materials and Methods

### 2.1. Plant Materials and Reagents

The freshly harvested *Ananas comosus* L. Merr peels were obtained from a local fruit stall at Taman Universiti, Johor Bahru. The plant was authenticated by a biologist, Dr. Mohd Firdaus Ismail, and the voucher specimen (MFI 0110/19) was deposited into the Biodiversity Unit of Universiti Putra Malaysia. The peels were thoroughly rinsed and dried in a ventilated oven (2 days) at 50°C. The desiccated peels were pulverized using a laboratory grinder followed by sifting through a 40−mesh sieve to obtain a uniformly sized powder. The peels were then stored in ziplock bags (4°C) until further use.

### 2.2. Chemicals and Reagents

Grapeseed oil (GSO) and extra virgin olive oil (OO) were purchased from Borges (Spain) and Basso (Italy), respectively. Cosmeceutical grade Tween 80 (T80) was bought from Lamberti S.P.A (Italy) while xanthan gum (XG) was procured from Deosen Biochemical (Ordos) Ltd., China. Cosmeceutical grade phenoxyethanol (Phy-Et) was purchased from Nacalao Tesque (Japan), while the red blossom fragrance oil was purchased from Luzi Fragrance Compounds (Johor, Malaysia). Other chemicals were obtained from the General Chemistry Laboratory of Faculty Science, Universiti Teknologi Malaysia. Deionized water was purified using the MilliQ® Direct 8 water system (Merck KGaA, Darmstadt, Germany).

### 2.3. Preparation of the *A. comosus* Peels Extract

The dried and finely ground peels (1 g) were transferred into a centrifuge tube containing a mixture of ethanol and water (50%, v/v) and homogenized (10, 000 rpm, 40 s) using the homogenizer IKA T18 Digital Ultra Turrax (Germany). The sample was then ultrasonically extracted using a 20 kHz ultrasonic generator (130 W, KH5200DB type, Kunshan ultrasonic instrument Co., Ltd., Jiangsu, China) using a 5 min sonication time (65% amplitude) at a constant temperature of 30°C ± 1°C. The sample was ultracentrifuged (6,000 rpm, 15 min), and the resultant supernatant was transferred into a conical flask (150 mL). The supernatants were combined, filtered through a Whatman filter paper No.1, and concentrated using a rotary evaporator (Cole Palmer, USA) at 40°C. The crude extract was lyophilized (24 h) and chilled (4°C) until further analysis.

### 2.4. Preparation of the Optimized Nano Cream

Triplicate optimal oil-in-water (O/W) nano creams were prepared by combining the low and high-energy methods: phase inversion temperature (PIT) and probe ultrasonication. Preparation of the oil phase and the aqueous phase was done separately, by which a mixture of OO (1%), GSO (12%), and T80 (12.63%) formed the oil phase, and distilled water (74.37%) and XG (2%) made up the aqueous phase. The oil and aqueous phases were heated to 70 ± 1°C, and the oil phase was added dropwise into a beaker containing the aqueous phase with stirring (600 rpm), followed by the addition of *Ac*PE (10%). Using a 20 kHz ultrasonic generator (130 W, KH5200DB type, Kunshan ultrasonic instrument Co., Ltd., Jiangsu, China), the coarse emulsion was ultrasonicated (5 min) with the simultaneous addition of Phy-Et (1%) and perfume oil (2%).

### 2.5. pH, Particle Size, and Polydispersity Index Monitoring for Accelerated Stability Test

The optimized nano creams used in this study were prepared by observing the physicochemical properties in our previous work [18]. Consequently, the nano creams were assessed weekly for accelerated stability tests for particle size, polydispersity index (PDI), and presence of phase separation under 6 weeks of storage at three different temperatures (4°C, 25°C, and 50°C). The particle size and PDI were determined simultaneously using the Zetasizer Nano ZSP instrument (Malvern Instruments, UK). Particle size, a vital parameter in this study, was monitored for 6 weeks to evaluate the destabilization phenomena related to coalescence and Ostwald ripening in the optimized nano creams under an extended storage duration. Meanwhile, the pH value of the optimized nano creams stored at 25°C for 6 weeks was checked at regular intervals weekly using the Delta 320 pH meter (Melter-Toledo, Schwerzenbach, Switzerland). All the above experiments were prepared in triplicate, and the results are presented as mean ± standard deviation.

### 2.6. Rate of Destabilization Mechanism

#### 2.6.1. Coalescence Rate

The coalescence rate of the optimized nano creams was assessed in terms of particle size over 6 weeks of storage at three different storage temperatures using the following equation :

(1) $$\begin{array}{c} \frac{1}{r^{2}}=\frac{1}{r_{0}^{2}}-\left(\frac{8\pi }{3}\right)\omega t. \end{array}$$

As shown in equation (1), *r* refers to the mean radius after time, and the term *r*_0_ represents the value at a time (s) *t* = 0. Meanwhile, *ω* refers to the frequency of rupture per unit of the film surface. This study plotted the graph of 1/*r*^2^ versus storage time (s) to assess the rate of coalescence in the optimized *Ac*PE. A linear relationship graph was predicted for the optimal nano cream influenced by the coalescence rate. All the above experiments were prepared in triplicate.

#### 2.6.2. Ostwald Ripening Rate

The Lifshitz–Slyosov–Wagner theory was used to determine the Ostwald ripening rate for the optimal nano creams under the influence of three different temperatures. Ostwald ripening occurs when the particle size of the system increases to a certain extent due to the diffusion of the oil phase into the aqueous phase. The Ostwald ripening rate was calculated as follows:

(2) $$\begin{array}{c} \omega =\frac{dr^{3}}{dt}=\frac{8}{9}\left[\frac{C\left(\infty \right)V_{m}D}{\rho RT}\right], \end{array}$$

where *ω* denotes the rupture frequency per unit film surface, *r* represents the average droplet radius over time, *t* refers to the storage time (s), and *C* (∞) refers to the bulk phase solubility. The term *V*_*m*_ is the molar volume of the internal phase, *D* denotes the diffusion coefficient of the dispersed phase in the continuous phase, and *ρ* is the density of the dispersed phase. The term *R* denotes the gas constant, and *T* is the absolute temperature. The Ostwald ripening rate of the optimized *Ac*PE nano creams was visualized by plotting a graph of *r*^3^ versus storage time (s) under the varying storage temperatures. All the above experiments were prepared in triplicate.

### 2.7. Microbiological and Heavy Metal Test Limit for Cosmetic Product

A cosmetic product intended for consumers must be safe and comply with the Malaysian cosmetic legislation guidelines for control of cosmetic products. Therefore, the safety evaluation of OPT-AcPE nano creams was evaluated using two different tests: microbial and heavy metal test limit as suggested by National Pharmaceutical Regulatory Agency (NPRA) Malaysia [19] (Appendix I). Both tests are explained further in the section below.

#### 2.7.1. Microbiological Test Limit

The microbiological test is vital for safety purposes as it can affect the stability and efficacy of products and identify any possible serious health risks to consumers [20]. Therefore, microbial analysis of the optimized *Ac*PE nano creams was performed using the standard method of analysis according to the Bacteriological Analytical Manual (Chapter 3: Microbiological Methods for Cosmetics) of the Food and Drug Administration (FDA) [21]. The tests included the detection of specific microorganisms and a total microbial count. The National Pharmaceutical Regulatory Agency (NPRA) has specified that the limit for total microbial count should not exceed 100 cfu/g [19]. Specific microorganisms designated to be the primary potential pathogens in a cosmetic formulation comprise *Staphylococcus aureus sp. (S. aureus)*, *Pseudomonas aeruginosa sp. (P. aeruginosa)*, and *Candida albicans sp. (C. albicans)* were used in the test. For accuracy, all the above experiments were prepared in triplicate.

#### 2.7.2. Heavy Metal Test Limit

Dermal exposure to heavy metal is deemed the most significant route since most products require a topical application [22]. Thus, this present reported heavy metal test observed four elements: arsenic (As), cadmium (Cd), mercury (Hg), and lead (Pb) in the optimized *Ac*PE nano creams. The As, Cd, and Pb were analyzed using in-house method LWI-MFF 021 based on the AOAC 968.08 (Sample preparation) and USEPA 6010B methods. Meanwhile, the presence of Hg was assessed using an in-house method, LWI-MFF 027, based on the USEPA 7473 using the Mercury Analyzer (Milestone, USA). All the above experiments were prepared in triplicate.

## 3. Results and Discussion

### 3.1. pH, Particle Size, PDI Monitoring for Accelerated Stability Test

**Figure 1:**
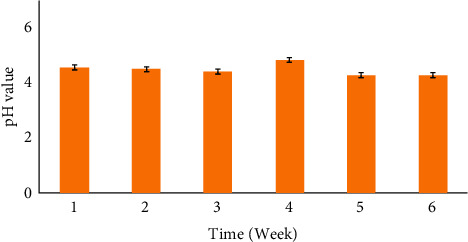
A time-course depicting the change in pH value of the optimized *Ac*PE nano cream at 25°C.

It is crucial to mention here that an accelerated stability test aims to examine the degradation of a sample under similar environments as those subjected to real-time (long-term) storage conditions, except for a slightly shorter storage duration [23]. This test has been a standard for pharmaceuticals for over 60 years, and it benefits the manufacturers to assess and predict the; (i) compatibility of ingredients and (ii) shelf life of formulations from the aspect of product development [24, 25]. Therefore, the accelerated stability on the optimized *Ac*PE nano cream was done according to Cosmetic Europe—The Personal Care Association, Personal Care Products Council (PCPC), and Brazil National Health Surveillance Agency (ANVISA) guidelines [25, 26].  Figure 1 illustrates the changes in pH of the *Ac*PE nanocream within 6-weeks of storage at 25°C. Notably, the samples' pH values maintained an acceptable range (pH 4–7) throughout the storage duration. In fact, a stable, skin-compatible pH of the optimized *Ac*PE nano cream (pH < 5) is crucial for alleviating the discomfort of skin problems such as dermatitis or impaired skin barrier [27, 28]. Also, Nigro et al. [29] and Roselan et al. [30] documented the same outcome in their copaiba oil and kojic monooleate nanoemulsions, registering the corresponding pH values of 4.60 and 5.75. Hence, the findings of this study supported the adequacy of the optimal nanocream as a topical application on the human skin.

**Figure 2:**
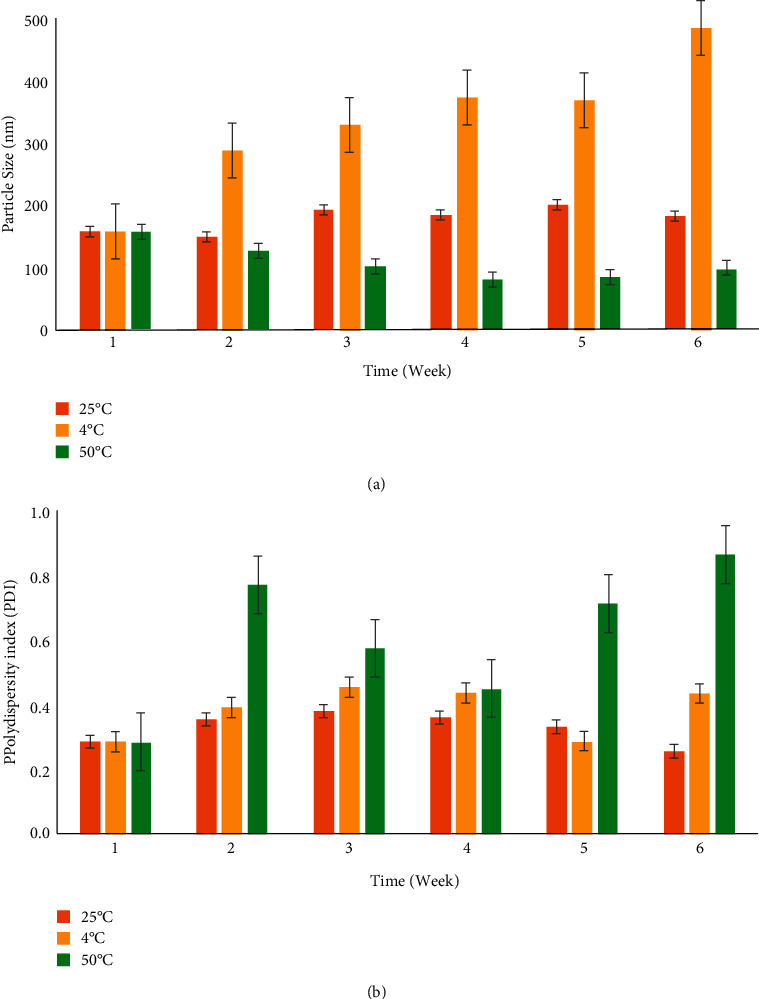
Time-course plots depicting the change in (a) particle size (nm) and (b) PDI of the optimized *Ac*PE nano cream under different storage temperatures (4°C, 25°C, and 50°C).

Figure 2 depicts the changes in particle size and PDI at 6 weeks of storage under varying temperatures. As seen in Figure 2(a), the particle size of the *Ac*PE nano cream stored at 25°C retained an average nanometre-range particle size (<200 nm) with good stability throughout the assessed duration.

However, it was expected that the particle size of the *Ac*PE nano cream to change as the storage temperature was elevated drastically. As seen in Figure 2(a), the particle size of the *Ac*PE nano cream stored at 4°C exhibited an increasing trend from 157.93 ± 1.88 to 485.53 ± 0.68 nm, but in the absence of visible creaming or phase separation. The growth in particle size seen here could be attributed to a destabilization phenomenon such as coalescence or Ostwald ripening. Literature has shown that larger particles are formed in a nanoemulsion system through coalescence, where particles collide and merge [31]. Contrariwise, the Ostwald ripening energetically promotes the condensation and aggregation of smaller particles into a bulk, which progressively increases the particle's size [32, 33]. Similar observations were also reported by Nejadmansouri et al. [34] and Roselan et al. [30].

Interestingly, the particle size of the optimized *Ac*PE nano cream reduced from 157.93 ± 1.88 to 98.19 ± 11.44 nm when stored at the highest temperature (50°C), accompanied by phase separation towards the end of the 6-week storage. Zhao et al. [35] attributed this outcome to the increased frequency of particles collision as the temperature increases. Usually, a system's elevated kinetic energy at a higher storage temperature usually exacerbates aggregation and increases particle size. However, this study's observation was contradictory. The opposite outcome seen here might be related to the *Ac*PE particles' surface not being fully covered by the surfactant (T80) during the formulation process. It was likely the consequence of an inadequate time interval of particle production and particle size determination. As a result, the T80 particles continue to penetrate and fully cover the *Ac*PE particle surfaces during storage, thus reducing particle size over a longer storage duration. A similar outcome was reported in recent studies by Akhoond Zardini et al. [36] and Borba et al. [37].

As seen in Figure 2(b), the optimized *Ac*PE nano cream remained monodispersed (<0.40) when stored at 4 and 25°C, indicating the long-term stability of the products [38, 39]. However, PDI values were seen to increase in week 2 for samples stored at 50°C. By week 4, the optimized *Ac*PE nano cream transformed from a monodispersed (0.286 ± 0.004) to a polydispersed system (0.865 ± 0.208). The outcome seen here concurred with the findings of Al-Maqtari et al. [40] that found the PDI value of *Pulicaria jaubertii extract* formulation stored at 25°C increased from 0.348 to 0.708 after 28 days. A plausible explanation for this might be water loss from the coalescence effect and dehydration of nonionic surfactant (T80) in the *Ac*PE nano cream. According to Mohammed et al. [41], water loss affects bonds between the ethylene oxide group and adjacent polyoxyethylene groups, presumably causing the emulsion's coalescence at the end of the storage period. As a matter of fact, the dehydration effect on T80 was influenced by the surfactant's molecular structure. To be precise, it was likely related to the separation of the ether-bonded oxygen atom and the deterioration in the hydrophilic properties of nonionic surfactants. This leads to the component being no longer water-soluble due to the weakened hydrogen bonds, thus explaining the instability of optimized *Ac*PE nano cream towards the end of the storage period [42].

Notwithstanding, the data also revealed the optimum particle size (<200 nm) and PDI (<0.40) of the *Ac*PE nano cream, proven by the absence of phase separation after 6 weeks of storage at 25°C. The system's stability seen here might be due to the synergistic interaction between the formulation's nonionic surfactant (T80) and thickener (XG). The adequate presence of T80 in the nano cream appears to induce steric stabilization by forming a physical barrier between the particles. This, in turn, reduces the systems' susceptibility to coalescence [43]. Besides, the addition of XG to the *Ac*PE nano cream further improves the systems' stability by increasing viscosity which encourages mechanical stabilization through reduced particle mobility [43, 44]. Hence, the acceptable storage temperature for the optimized *Ac*PE nano cream was shown to be 25°C.

### 3.2. Coalescence Rate

**Figure 3:**
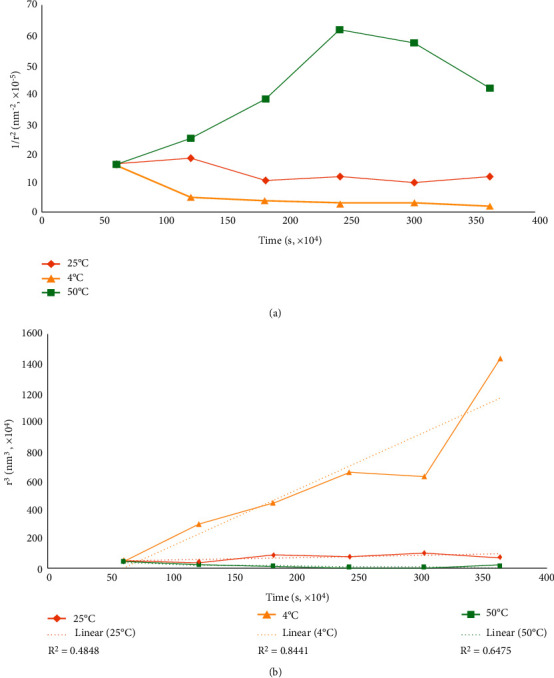
The (a) coalescence rate and (b) Ostwald ripening rate of the optimized *Ac*PE nano cream at different temperatures (4°C, 25°C, and 50°C) for 6 weeks of storage.

Coalescence is a destabilization process where two or more particles combine to give a larger mean particle size in a nanoemulsion [31, 32]. The rate of coalescence of the optimized *Ac*PE nanocream in relation to the change in storage temperature can be visualized by plotting a graph of 1/*r*^2^ versus storage time (seconds) [45], where Kundu et al. [46] described that instability due to coalescence produces a linear graph. However, the nonlinear relationship between particle size over storage time (Figure 3(a)) in the optimized *Ac*PE formulation signified that particle size changes under the three different storage temperatures were not coalescence related. Likewise, studies by Roselan et al. [30] achieved similar nonlinear graphs for the nonrelated coalescence destabilization on their formulated nanoemulsions.

Meanwhile, a significant reduction in particle size with a concomitant nonlinear pattern of the optimized *Ac*PE nano cream at 50°C ruled out the coalescence-related instability. Thus, we postulated that the phase separation seen in the 50°C stored samples at the end of 6-week storage was not due to the water loss. However, Galvão et al. [47] believed that their destabilized O/W pepper nanoemulsion at 37°C after 60 days was due to a dehydrated system. Instead, a considerable size increase for *Ac*PE samples stored at 4°C pointed to the possible contribution of the Ostwald ripening in the system. Thus, this study further assessed the possibility of this phenomenon in the following subsection.

### 3.3. Ostwald Ripening Rate

Ostwald ripening is a process where particles become larger at the expense of the smaller ones due to the disappearance and diffusion of smaller droplets into the bulk [45]. This destabilizing phenomenon sees the dispersed phase molecules diffuse through the continuous phase in the colloidal system. Ostwald ripening-related changes produce larger molecules that are energetically more favorable. From an exclusively kinetic perspective, larger clusters are thermodynamically favored. The effect of Ostwald ripening on the *Ac*PE nano cream is best visualized from the linear relationship of *r*^3^ versus storage duration (seconds) [48]. Aside from the linearity of the graph, the Ostwald ripening effect on the system is manifested in the graph's linear regression (*R*^2^ > 0.8) [47].

In Figure 3(b), the *Ac*PE nano cream stored at 4°C exhibited an appreciable Ostwald ripening contribution, which elevated the particle size during storage. A deviation from linearity at the beginning of the graph could be attributed to initial coalescence. However, the *Ac*PE nano cream later became destabilized by Ostwald ripening when storage duration was factored into the investigation . In reference, Kundu et al. [46] and Jaslina et al. [45] also uncovered similar linear graphs that supported Ostwald ripening being the destabilizing phenomenon. Also, a high *R*^2^ value (0.8441) supported that Ostwald ripening contributed to the elevated particle size in the *Ac*PE nano cream.

However, the nonlinear correlation in *Ac*PE nano cream samples stored at 50°C (*R*^2^ = 0.6475) ruled out Ostwald ripening as the destabilizing phenomenon. Presumably, the outcome seen here was related to the dehydration of T80 that caused the samples' phase separation towards the end of the storage. In fact, the storage temperature being close to the production temperature contributed to this unfavorable outcome. On top of that, high temperatures elevate particles' kinetic energy and movement [30], which promotes the nanoemulsion's instability, elevating the nanoemulsion's PDI. This series of changes, thus, explains the phase-separated *Ac*PE nano cream stored at 50°C without the increase in particle size after 6 weeks.

In retrospect, this study's outcome was consistent with a previous report by Ravera et al. [49] on nanoemulsions becoming destabilized, an inter-related phenomenon that impacts one another during storage. Conversely, Ostwald ripening did not affect the optimal *Ac*PE nano cream stored at 25°C. The nonlinear data distribution alongside the low *R*^2^ value (0.4848) corroborated the observed outcome. Jiménez-Rodríguez et al. [50] explained that the stability of a nanoemulsion increases with a smaller particle size as the colloidal system behaves correspondingly to Brownian motion, reducing the gravitational separation force. This factor also correlates to lower particles' kinetic energy at low temperatures, minimizing the particles' mobility in the system [51]. Accordingly, the study outcome agrees with the stability data obtained in sub-section 3.1, proving that 25°C is a suitable storage temperature for the optimized *Ac*PE nano cream.

### 3.4. Microbiological Test Limit

There is the possibility of unwanted growth of microorganisms in cosmetic products under an extended shelf life. It is due to the presence of water and other nutrients in the formulation [20]. Therefore, the microbial test is compulsory in cosmetic products to ensure safety and stability within their shelf-life [52]. The above-said test conducted by this study revealed that both bacteria and fungi were not detected as seen in fewer than 10 cfu/g or equivalent to 1000 cfu/g in the *Ac*PE nano cream samples. Also, microorganisms *viz. S. aureus*, *P. aeruginosa*, and *C. albicans* were absent in the tested samples. These test microorganisms are specified in the NPRA guidelines under the microorganisms of concern in cosmetic samples acquired through postmarket surveillance activities [19].

The inhibited growth of microorganisms in the *Ac*PE nano cream was due to the incorporated preservative (Phy-Et) in the formulation, coupled with the high concentration of polyphenolic compounds in *Ac*PE, as reported in our previous work [18]. The two bioactive ingredients thus augmented the antimicrobial strength of the *Ac*PE nano cream [53]. Also, Gram-positive bacteria are more vulnerable to polyphenols than Gram-negative bacteria due to the former's outer membrane deficiency. This causes polyphenolic compounds to diffuse easily through the former's cell wall, destabilizing the structure and eventually cell death [54]. Hence, the study's findings affirmed the microbial safety of the *Ac*PE nano cream for topical application.

### 3.5. Heavy Metal Test Limit

Dermal absorption is among the pathway of heavy metal exposure following the close contact of the product with the skin [22]. The deliberate use or unintentional incorporation of heavy metal in a product might endanger consumers, which is not tolerated in halal cosmetics, as it is against the halal concept of Shariah law [55]. However, an internationally harmonized system remains unavailable for a cosmetic product's safe permissible heavy metals limit. Accordingly, the study's limits for heavy metals followed the Malaysian guidelines for the control of cosmetic products, complying with the ASEAN Cosmetic Directive (ACD). Based on Annex 1 (Part 6) in the guidelines, each type of heavy metal has a specific allowable limit. The strict adherence to these limits is a requisite for a good manufacturing process [19].

Based on the study findings, the topically administered optimized *Ac*PE nano cream could preserve the good quality of the skin without posing any potential health hazard to consumers. This was affirmed by the low levels of heavy metals of concern, namely, As, Cd, Pb, and Ag (0.1 to 0.5 mg/kg). The presence of As, Cd, Pb, and Ag in the optimized *Ac*PE nano cream was well below their allowable limits of 5.0, 20.0, and 1.0 mg/kg, respectively. It could be construed that the *Ac*PE nano cream is safe from heavy metals contamination, and the formulation was done in a clean environment.

## 4. Conclusion

The optimal nanocream was not influenced by coalescence, but it was under a significant influence of the Ostwald ripening destabilization phenomenon at 4°C at 6 weeks of storage. The study confirmed that 25°C was the optimum storage temperature for the optimized *Ac*PE nano cream, and the cream retained nano-range particle size (<200 nm) and monodisperse system (<0.40). The findings also affirmed that the *Ac*PE nano cream was safe and free from microbial and heavy metal contamination. These attributes thus conveyed the nanoemulsion's suitability for topical application on the skin, fulfilling the Malaysian cosmetic guidelines. In a nutshell, the optimized *Ac*PE nano cream developed in this study, which focuses on naturally derived plant active ingredients, has potential application in the cosmeceutical industry.

## Data Availability

The datasets used during the study are available from the corresponding author on reasonable request.

## Supplementary Materials

Supplementary Materials Informed consent form for sensory evaluation.
