# Supplementary figures and images for: Ananas comosus Peels Extract as a New Natural Cosmetic Ingredient: Oil-in-Water (O/W) Topical Nano Cream Stability and Safety Evaluation
Source: Evid Based Complement Alternat Med. 2022 May 12;2022:2915644. doi: 10.1155/2022/2915644 (PMC9119789; doi:10.1155/2022/2915644)

#
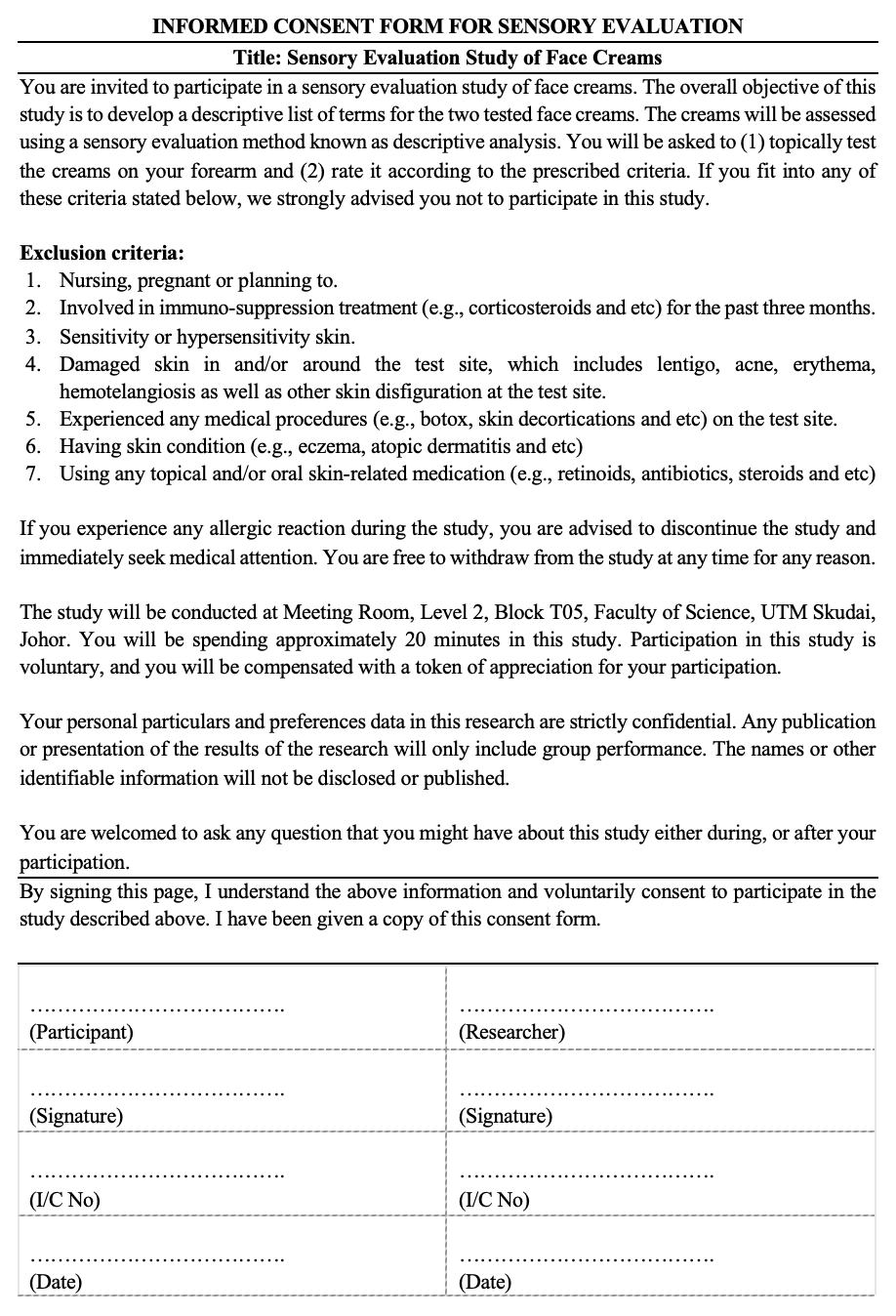
Supplementary File

Supplement: Supplementary Materials — Informed consent form for sensory evaluation. [file 2915644.f1.docx]
